# Supplementary material for: Giant Faraday rotation in atomically thin semiconductors
Source: Nat Commun. 2024 Apr 10;15:3082. doi: 10.1038/s41467-024-47294-5 (PMC11006678; doi:10.1038/s41467-024-47294-5)
Supplement: Supplementary file 1 — Supplementary information [file 41467_2024_47294_MOESM1_ESM.pdf]

Supplementary Information

Giant Faraday Rotation in Atomically Thin Semiconductors

Benjamin Carey[1,2], Nils Kolja Wessling[1,3], Paul Steeger[1], Robert Schmidt[1], Steffen Michaelis de Vasconcellos[1], Rudolf Bratschitsch[1,*], and Ashish Arora[1,4,*]

[1]Institute of Physics and Center for Nanotechnology, University of Münster, Wilhelm-Klemm-Strasse 10, 48149 Münster, Germany
[2]School of Mathematics and Physics, The University of Queensland, St Lucia, Queensland 4067, Australia
[3]Institute of Photonics, Department of Physics, University of Strathclyde, 99 George Street, G1 1RD Glasgow, UK
[4]Department of Physics, Indian Institute of Science Education and Research, Dr. Homi Bhabha Road, 411008 Pune, India

**Email: ashish.arora@iiserpune.ac.in, rudolf.bratschitsch@uni-muenster.de*

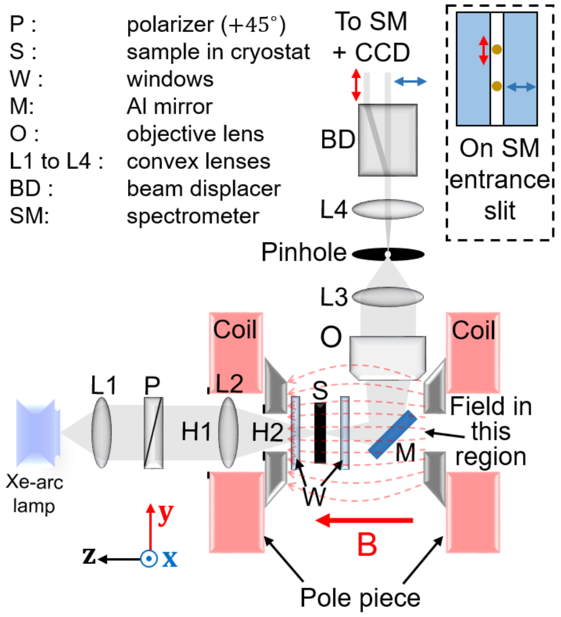

Figure S1. Experimental Setup. Broadband light from a Xe arc lamp is collimated after passing through the lens L1, and is linearly polarized at 45° to the $\hat{\mathbf{x}}$-axis using a polarizer P. It is focused on the sample S using a lens L2. S is mounted on the copper cold finger of a continuous-flow liquid He cryostat. The sapphire windows W maintain the vacuum in the cryostat, while letting the light pass through. A front-polished aluminum mirror reflects the light towards $\hat{\mathbf{y}}$ direction which is collimated by a 10x objective lens O. Lens L3 focuses it on a pinhole of 20 µm diameter, while L4 collimates it again. The light is spatially separated into two linear polarization components using a beam displacer BD. This is wavelength dispersed after passing through a 0.3 m focal length spectrometer, and is detected using a Peltier-cooled CCD.

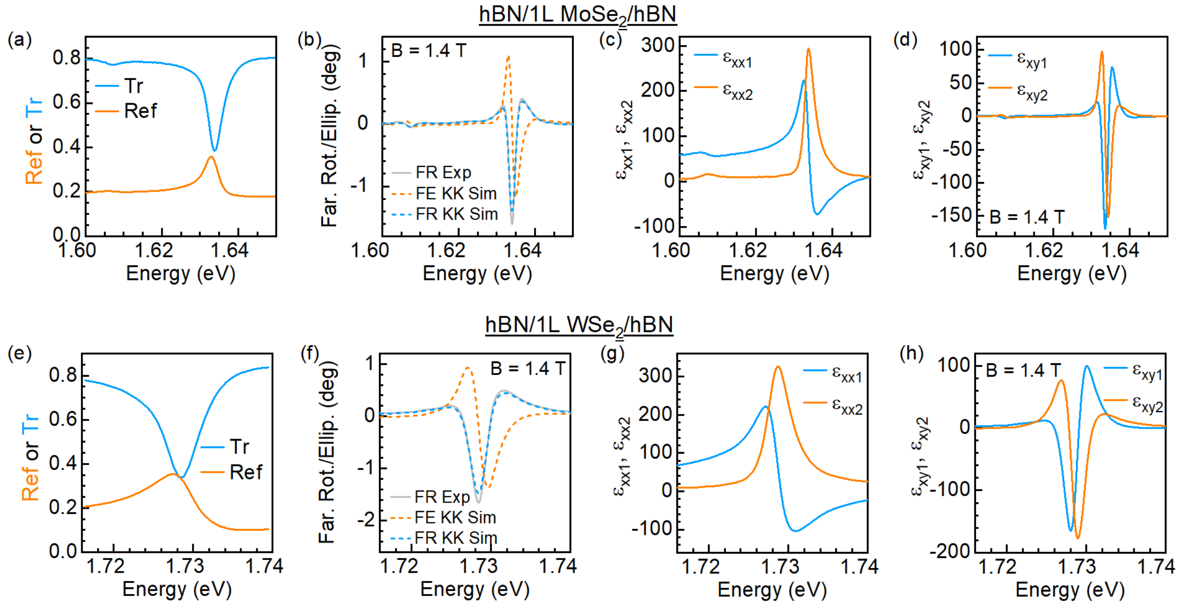

Figure S2. Determination of the complex dielectric tensor of the hBN-encapsulated TMDC monolayers. In (a) and (e), solid curves show optical reflectance and transmittance spectra of hBN-encapsulated $MoSe_2$ and WSe_2 monolayers, respectively. In (b) and (f), solid lines are the experimentally measured Faraday rotation (FR) spectra of the two materials, respectively, under a magnetic field of 1.4 T at a temperature T = 10 K. Dashed orange lines are the Faraday ellipticity (FE) spectra calculated using a Kramers-Kronig (KK) analysis, using the solid lines as inputs. To test the validity of the calculation, a KK analysis is again applied with calculated FE spectra as inputs, and FR spectra as computed (dashed blue lines). The computed FR spectra (dashed blue lines) are in a reasonable agreement with the respective experimental measurements (solid lines), giving us confidence in our calculation. (c) and (g) display the calculated real and imaginary parts of the diagonal elements of the dielectric tensor, while (d) and (h) show the off diagonal elements, for the two materials, respectively.

Microscopic explanation of the Faraday rotation in semiconducting transition metal dichalcogenides.

In the main text, we argue that the excitonic Faraday rotation in semiconducting transition metal dichalcogenides (TMDCs) such as MoS_2, $MoSe_2$, WS_2 and WSe_2 originates due to a combined effect of a giant exciton oscillator strength and a large exciton g factor. Here we explain the origins of the Zeeman splitting in TMDCs in a more detailed fashion on microscopic levels following literature[1–6], by using a $MoSe_2$ monolayer as an example. The optical transitions relevant to the present work take place at the $K^{\pm}$ points ('valleys') of the hexagonal Brillouin zone of the TMDC monolayer[3,4]. Due to an absence of inversion symmetry, and a strong spin-orbit interaction in a TMDC monolayer, the top (bottom) of the valence (conduction) bands at the K^+ and K^- points are associated with opposite signs of magnetic moments[1,5–8](Fig. S3). Due to this, the optical transitions at the K^+ and K^- points possess opposite helicity of circular polarization i.e. σ^+

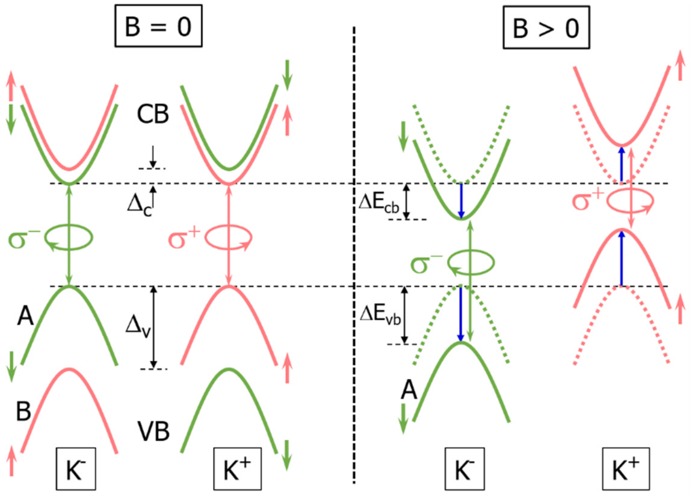

Figure S3. Band structure around $K^{\pm}$ points under a magnetic field. *Left panel: Spin-orbit-split conduction and valence bands (CB and VB) at the $K^{\pm}$ points of the Brillouin zone of a monolayer TMDC such as $MoSe_2$, in the absence of a magnetic field. Δ_c and Δ_v are the spin-orbit splitting in CB and VB. Right panel: Coupling of spin and atomic orbital magnetic moments at the top of the VB and the bottom of the CB with the external magnetic field resulting in the energetic shift of bands depicted by blue arrows. This lifts the energetic degeneracy of the $\sigma^{\pm}$ transitions at the $K^{\pm}$ points. Valley Zeeman splitting is given as $\Delta E = 2(\Delta E_{cb} - \Delta E_{vb}) \sim g_X \mu_b B$ where g_X is the exciton g factor.*

and σ⁻ respectively[6,9–11]. In the absence of a magnetic field, the two transitions are energetically degenerate[5] (Fig. S3). However, in the presence of a magnetic field, the energies of the conduction and valence bands involved in the exciton transition are modified through magnetic coupling with the magnetic moments. Since the two valleys have opposite magnetic moments, they couple with the magnetic field in an opposite manner, moving bands at the K^+ and K^- points in opposite directions (Fig. S3). This results in a breaking of degeneracy of the $\sigma^\pm$ optical transitions i.e. a *'Valley Zeeman splitting'* of the exciton, $\Delta E = E_{\sigma^+} - E_{\sigma^-} = 2(\Delta E_{cb} - \Delta E_{vb}) \sim g_X \mu_b B$ where g_X is the exciton g factor[5–7,12]. Experimentally, the g factors of the excitons and trions in monolayer TMDCs are observed to be approximately equal to -4[5,6,12]. Direct microscopic calculations of the exciton g factor on the basis of *GW*-BSE *ab initio* theory predicts the values of the exciton g factors very close to -4,[1]. Other groups have also calculated the interband-transition g-factors of similar magnitudes without including excitonic effects[8,13,14].

Faraday rotation of the linearly polarized light passing through a TMDC arises due to valley Zeeman splitting as follows. The electric fields of the $\sigma^\pm$ circularly polarized components of the incident linear polarization (assuming along $\hat{\mathbf{y}}$ for the sake of argument) $\vec{\mathcal{E}}(t) \propto \sin\left(\frac{E_l}{\hbar}t\right)\hat{\mathbf{y}}$ interacting with the $K^\pm$ valleys of the TMDC can be proportionately represented in the form of Jones vectors as follows:

$$\vec{\mathcal{E}}_{K^\pm}(t) \propto \begin{pmatrix} \mp\cos\left(\frac{E_{\sigma^\pm}}{\hbar}t\right) \\ \sin\left(\frac{E_{\sigma^\pm}}{\hbar}t\right) \end{pmatrix} \tag{S6}$$

A superimposed electric field of light after passing through the material is given as

$$\vec{\mathcal{E}}(t) \propto \sin\left(\frac{E_{\sigma^+}+E_{\sigma^-}}{2\hbar}t\right) \begin{pmatrix} \sin\left(\frac{\Delta E}{2\hbar}t\right) \\ \cos\left(\frac{\Delta E}{2\hbar}t\right) \end{pmatrix} \sim \sin\left(\frac{E_l}{\hbar}t\right) \left[\frac{\Delta E}{2\hbar}t\ \hat{\mathbf{x}} + \hat{\mathbf{y}}\right] \text{ for small } \Delta\mathrm{E} \tag{S7}$$

In the absence of an external magnetic field, $\Delta E = 0$. Therefore, $\vec{\mathcal{E}}(t) \propto \sin\left(\frac{E_l}{\hbar}t\right)$ with $E_l = E_{\sigma^+} = E_{\sigma^-}$ and there is no rotation of the electric field. However, in the presence of the magnetic field, the valley Zeeman splitting of the exciton ($\Delta E \neq 0$) results in a Faraday rotation of $\frac{\pi}{2} - \cot^{-1}\left(\frac{\Delta E}{2\hbar}t\right)$ towards the $\hat{\mathbf{x}}$.

References.

1. Deilmann, T., Krüger, P. & Rohlfing, M. Ab Initio Studies of Exciton g Factors: Monolayer Transition Metal Dichalcogenides in Magnetic Fields. *Phys. Rev. Lett.* **124**, 226402 (2020).
2. Kormányos, A. *et al.* k·p theory for two-dimensional transition metal dichalcogenide semiconductors. *2D Mater.* **2**, 022001 (2015).
3. Wang, G. *et al.* Colloquium : Excitons in atomically thin transition metal dichalcogenides. *Rev. Mod. Phys.* **90**, 021001 (2018).
4. Mak, K. F., Xiao, D. & Shan, J. Light–valley interactions in 2D semiconductors. *Nat. Photonics* **12**, 451–460 (2018).
5. Koperski, M. *et al.* Orbital, spin and valley contributions to Zeeman splitting of excitonic resonances in $MoSe_2$, WSe_2 and WS_2 Monolayers. *2D Mater.* **6**, 015001 (2018).
6. Arora, A. Magneto-optics of layered two-dimensional semiconductors and heterostructures : Progress and prospects. *J. Appl. Phys.* **129**, 120902 (2021).
7. Arora, A. *et al.* Valley Zeeman Splitting and Valley Polarization of Neutral and Charged Excitons in Monolayer $MoTe_2$ at High Magnetic Fields. *Nano Lett.* **16**, 3624–3629 (2016).
8. Woźniak, T., Faria Junior, P. E., Seifert, G., Chaves, A. & Kunstmann, J. Exciton g factors of van der Waals heterostructures from first-principles calculations. *Phys. Rev. B* **101**, 1–29 (2020).
9. Mak, K. F., He, K., Shan, J. & Heinz, T. F. Control of valley polarization in monolayer MoS_2 by optical helicity. *Nat. Nanotechnol.* **7**, 494–498 (2012).
10. Zeng, H., Dai, J., Yao, W., Xiao, D. & Cui, X. Valley polarization in MoS_2 monolayers by optical pumping. *Nat. Nanotechnol.* **7**, 490–493 (2012).
11. Sallen, G. *et al.* Robust optical emission polarization in MoS_2 monolayers through selective valley excitation. *Phys. Rev. B* **86**, 081301 (2012).
12. Srivastava, A. *et al.* Valley Zeeman effect in elementary optical excitations of monolayer WSe_2. *Nat. Phys.* **11**, 141–147 (2015).
13. Xuan, F. & Quek, S. Y. Valley Zeeman effect and Landau levels in two-dimensional transition metal dichalcogenides. *Phys. Rev. Res.* **2**, 033256 (2020).
14. Förste, J. *et al.* Exciton g-factors in monolayer and bilayer WSe_2 from experiment and theory. *Nat. Commun.* **11**, 4539 (2020).
